# Supplementary material for: Synergizing language learning: SmallTalk AI In industry 4.0 and Education 4.0
Source: PeerJ Comput Sci. 2024 Jan 31;10:e1843. doi: 10.7717/peerj-cs.1843 (PMC10909197; doi:10.7717/peerj-cs.1843)
Supplement: Supplemental Information 4 [file peerj-cs-10-1843-s004.docx]

Technology Acceptance Model Questionnaire

Perceived Usefulness (PU)

| S.No. | Context | Questions | Strongly agree | Agree | Neutral | Disagree | Strongly Disagree |
| --- | --- | --- | --- | --- | --- | --- | --- |
| 1 | Perceived Usefulness (PU1) | SmallTalk enable me to accomplish speaking more quickly for innovations |  |  |  |  |  |
| 2 | Perceived Usefulness (PU2) | SmallTalk has improved the quality of innovations within fluency |  |  |  |  |  |
| 3 | Perceived Usefulness (PU3) | SmallTalk make it easier to innovate vocabulary |  |  |  |  |  |
| 4 | Perceived Usefulness (PU4) | SmallTalk has improved interaction productivity |  |  |  |  |  |
| 5 | Perceived Usefulness (PU5) | The use of SmallTalk increases the effectiveness of performing speaking tasks. |  |  |  |  |  |
| 6 | Perceived Usefulness (PU6) | Using SmallTalk give me access to a lot of pronunciation information |  |  |  |  |  |

Perceived Ease of Use (PEU)

| S.No. | Context | Questions | Strongly agree | Agree | Neutral | Disagree | Strongly Disagree |
| --- | --- | --- | --- | --- | --- | --- | --- |
| 1 | Perceived Ease of Use (PEU1) | My interaction with SmallTalk in AI process has been clear and understandable |  |  |  |  |  |
| 2 | Perceived Ease of Use (PEU2) | Using SmallTalk enable me to have more accurate information on speaking |  |  |  |  |  |
| 3 | Perceived Ease of Use (PEU3) | Learning the speaking skills to operate with SmallTalk was easy for me |  |  |  |  |  |
| 4 | Perceived Ease of Use (PEU4) | The use of SmallTalk for speaking activities does not confuse me |  |  |  |  |  |
| 5 | Perceived Ease of Use (PEU5) | SmallTalk is easy to navigate |  |  |  |  |  |
| 6 | Perceived Ease of Use (PEU6) | Overall, SmallTalk is easy to use |  |  |  |  |  |

Behavioural Intention to Use (BIU)

| S.No. | Context | Questions | Strongly agree | Agree | Neutral | Disagree | Strongly Disagree |
| --- | --- | --- | --- | --- | --- | --- | --- |
| 1 | Behavioural Intention to Use (BIU1) | I intend to continue using SmallTalk for learning speaking activities |  |  |  |  |  |
| 2 | Behavioural Intention to Use (BIU2) | I intend to frequently use SmallTalk to perform my speaking tasks |  |  |  |  |  |
| 3 | Behavioural Intention to Use (BIU3) | Assuming I have access to SmallTalk for the innovation process, I intend to adopt it |  |  |  |  |  |
| 4 | Behavioural Intention to Use (BIU4) | Use SmallTalk more than any alternative applications |  |  |  |  |  |
| 5 | Behavioural Intention to Use (BIU5) | Use SmallTalk because of flexible time |  |  |  |  |  |
| 6 | Behavioural Intention to Use (BIU6) | Use SmallTalk because of the current trend in Industry 4.0 |  |  |  |  |  |
